# Danger of Spreading Disease by Books

**Published:** 1883-09

**Authors:** 


### Danger of Spreading Disease by Books.

The *Lancet*, quoting the alleged communication of yellow fever to an official in Paris, through a dispatch from Brazil; says circulating libraries are common sources of peril. It would be difficult to imagine a more powerful medium for conveying disease than books. Organic particles carrying infection may lie for weeks, months, or years between the pages of a bound book, to be dislodged by some susceptible person handling it. Measles, scarlet fever, diptheria, ordinary "sore-throat," whooping cough, bronchitis, (perhaps phthisis,) and other chest affections and some skin diseases, are most easily communicated by this means. Books cannot be disinfected without injury, and hence should be destroyed after use by those suffering with the class of diseases mention ed. Dispatches and letters come under the same category.

